# Optimal parameters for clinical implementation of breast cancer patient setup using Varian DTS software

**DOI:** 10.1120/jacmp.v13i3.3752

**Published:** 2012-05-10

**Authors:** Sook Kien Ng, Piotr Zygmanski, Andrew Jeung, Hassan Mostafavi, Juergen Hesser, Jennifer R. Bellon, Julia S. Wong, Yulia Lyatskaya

**Affiliations:** ^1^ Department of Radiation Oncology Brigham and Women's Hospital & Dana Faber Cancer Institute, Harvard Medical School Boston MA 02115 USA; ^2^ Department of Radiation Oncology, Mannheim Medical Centre University of Heidelberg Mannheim Germany; ^3^ Varian Medical Systems Inc. Palo Alto CA 94304‐1030 USA

**Keywords:** digital tomosynthesis, patient setup, shift‐and‐add algorithm, triangulation

## Abstract

Digital tomosynthesis (DTS) was evaluated as an alternative to cone‐beam computed tomography (CBCT) for patient setup. DTS is preferable when there are constraints with setup time, gantry‐couch clearance, and imaging dose using CBCT. This study characterizes DTS data acquisition and registration parameters for the setup of breast cancer patients using nonclinical Varian DTS software. DTS images were reconstructed from CBCT projections acquired on phantoms and patients with surgical clips in the target volume. A shift‐and‐add algorithm was used for DTS volume reconstructions, while automated cross‐correlation matches were performed within Varian DTS software. Triangulation on two short DTS arcs separated by various angular spread was done to improve 3D registration accuracy. Software performance was evaluated on two phantoms and ten breast cancer patients using the registration result as an accuracy measure; investigated parameters included arc lengths, arc orientations, angular separation between two arcs, reconstruction slice spacing, and number of arcs. The shifts determined from DTS‐to‐CT registration were compared to the shifts based on CBCT‐to‐CT registration. The difference between these shifts was used to evaluate the software accuracy. After findings were quantified, optimal parameters for the clinical use of DTS technique were determined. It was determined that at least two arcs were necessary for accurate 3D registration for patient setup. Registration accuracy of 2 mm was achieved when the reconstruction arc length was > 5° for clips with HU ≥ 1000°; larger arc length (≥ 8°) was required for very low HU clips. An optimal arc separation was found to be ≥ 20° and optimal arc length was 10°. Registration accuracy did not depend on DTS slice spacing. DTS image reconstruction took 10–30 seconds and registration took less than 20 seconds. The performance of Varian DTS software was found suitable for the accurate setup of breast cancer patients. Optimal data acquisition and registration parameters were determined.

PACS numbers: 87.57.‐s, 87.57.nf, 87.57.nj

## I. INTRODUCTION

Accurate patient setup during radiotherapy treatment is essential. However, it is particularly challenging when treating deformable organs, such as the breast.^(^ ^1^ ^,^ ^2^ ^)^ One solution for improving the setup of breast cancer patients is using surrogates such as surgical clips in the vicinity of the tumor cavity.^(^ ^3^ ^–^ ^6^ ^)^ Breast cancer patient setup using surgical clips with kV radiography has been demonstrated to be beneficial.^(^ ^6^ ^,^ ^7^ ^)^ However, the visibility of the clips in orthogonal kV radiographs strongly depends on the type of clip used (density, size, and material) and distance from the rib cage, the gantry orientation, and the appearance of overlying structures/hardware.^(^ ^8^ ^)^ Our earlier study^(^ ^8^ ^)^ showed that small clips (Horizon ligating clips, Teleflex Medical, Research Triangle Park, NC) and those that were placed close to the chest wall are less detectable in a kV radiograph. In addition, the clip's signal might be lost among more heterogeneous tissue, ribs, and immobilization hardware that are present in the background, resulting in failure of clip localization. Other complicating factors include clips that overlap along the beam projectory, high‐density structures (ribs or hardware), and scatter from the patient's body that creates too much noise for visualization of the clips. Similar findings were reported by Thomas et al.^(^ ^3^ ^)^ In addition, our clinical experience has shown that rotations and translations can be difficult to distinguish using only two orthogonal planar images. Therefore volumetric images are desired for accurate patient setup. When clip localization with orthogonal kV radiographs is not feasible, cone‐beam CT (CBCT) may be used.^(^ ^9^ ^–^ ^11^ ^)^ However, there are two major drawbacks associated with the use of CBCT for a breast cancer patient's setup: possible collision between the gantry head and the patient or couch, and relatively high imaging dose to the whole thorax.^(^ ^12^ ^–^ ^16^ ^)^ In another earlier study,^(^ ^15^ ^)^ depending on the placement of the imaging isocenter, five of eight breast cancer patients (63%) treated in the supine position were at risk of potential gantry collision during imaging. In addition, a restraint system, if used, might increase the occurrence of gantry collision as the restraint system would extend beyond the patient's body.

Recently, digital tomosynthesis (DTS) was evaluated as an alternative to CBCT.^(^ ^8^ ^,^ ^17^ ^–^ ^21^ ^)^ DTS has been shown to greatly reduce imaging dose, acquisition time, and the possibility of collisions. According to measurements made using a thorax phantom with the isocenter positioned at the center of the phantom,^(^ ^14^ ^)^ the DTS imaging dose ranged from less than 0.1 to 2.5 cGy compared to 3.8 to 6.2 cGy from the CBCT imaging. The DTS imaging technique for patient setup verification has been shown to be feasible for various disease sites such as head and neck,^(^ ^18^ ^,^ ^19^ ^,^ ^21^ ^)^ liver,^(^ ^18^ ^)^ prostate,^(^ ^18^ ^)^ lung,^(^ ^19^ ^)^ abdomen,^(^ ^19^ ^)^ and breast.^(^ ^22^ ^)^ While the feasibility of DTS was demonstrated for the treatment setup of patients with breast cancer, there are no reports of using any commercial software in the clinical setting for this purpose. In the present study, we determined the optimal acquisition and registration parameters using the Varian OBI system for data acquisition and nonclinical Varian DTS software for DTS reconstruction and registration.

## II. MATERIALS AND METHODS

### A. Study design

Varian DTS software (Varian Medical Systems, Palo Alto, CA) consists of three separate modules: (1) reconstruction of treatment DTS images from treatment CBCT projections, (2) reconstruction of reference DTS images from the planning CT, and (3) registration of DTS images to provide 3D shifts. The study was performed on images of two phantoms and ten patients.

Treatment DTS images were reconstructed from acquired CBCT projections, while reference DTS images were reconstructed from the planning CT using various parameters. The parameters used included arc lengths, number of arcs, angular separation between two arcs, arc orientations, and reconstruction slice spacing. An automated cross‐correlation match was performed on DTS image pairs. Each pair consisted of treatment and reference DTS images reconstructed using the same arc orientation. The match result of two or more short DTS arcs was triangulated to produce a 3D shift with improved accuracy. The obtained shift was compared to the shift determined from CBCT‐to‐CT registration performed using clinical software (Offline Review, Varian Medical Systems, Palo Alto, CA), which served as the baseline. The difference between the two shifts was used as an accuracy measure to evaluate the DTS software. The accuracy of the registration performed in Offline Review was of the order of 1−2 mm. This is consistent with the results of the study of CBCT‐based breast cancer patient setup by White et al.^(^ ^10^ ^)^ Considering such issues as calibration procedure, slice thickness, and pixel size, the inherent accuracy of the CBCT was estimated to be about 1 mm. As it is the best imaging modality available to us at present, we consider CBCT to be the “gold standard”.

#### A.1 Phantom data

A planning CT and CBCT of a rectangular phantom and a breast phantom were acquired. The rectangular phantom was a plastic container $\left(12\times \;20\;\times \;4\;{\text{cm}}^{3}\right)$ filled with a water‐equivalent material with five surgical clips $\left(M-11.5,\;1\;\times \;4\;{\text{mm}}^{2}\right)$ inserted in the phantom. The breast phantom was a breast mold filled with a water‐equivalent material in which five surgical clips of the same type were inserted. These clips were used for clip‐based registration purposes. The simplicity of the phantoms allowed for software evaluation without confounding interference from heterogeneous anatomical structures.

#### A.2 Patient data

Ten breast cancer patients (with 16 corresponding CBCT scans) were enrolled in this study under an IRB‐approved protocol. Planning CT and CBCT images were acquired for each patient per standard clinical practice. Each patient had 2–11 surgical clips implanted in the vicinity of the tumor bed. Depending on the distribution of the clips, only 2–7 clips were included within the final DTS volume reconstructed for registration. Characteristics of the clips used for the registration are listed in Table 1.

**Table 1:** Characteristics of clips and DTS arcs used in DTS image registrations.

*Patient*	*# of Clips Reconstructed*	*HU of Clips*	*Diameter of Clips*	*Length of Clips*	*Site*	$\theta_{c}$ *of 2 DTS Arcs*
1	2	400–600	2.5 mm	5.0 mm	LB	305°; 325°
2	5	1000–1350	3.0 mm	6.0 mm	RB	245°; 265°
3	2	1000–1400	3.0 mm	6.0 mm	RB	225°; 245°
4	4	400–600	2.5 mm	5.0 mm	RB	205°; 225°
5	3	1200–1500	3.0 mm	6.5 mm	LB	315°; 335°
6	4	1300–1500	3.0 mm	6.0 mm	LB	315°; 335°
7	5	1300–1500	3.0 mm	6.0 mm	LB	315°; 325°
8	3	600–1000	2.5 mm	5.0 mm	LB	305°; 325°
9	1	1300–1500	3.0 mm	6.0 mm	LB	315°; 335°
10	7	800–1000	2.5 mm	6.0 mm	LB	305°; 325°

Characteristics of the clips and the DTS arcs used in the registration of DTS images. Two short‐arc DTS images were used for triangulation in each data set. θc is the center angle of a particular arc orientation. LB=left breast, RB=right breast.

### B. DTS image reconstruction

Treatment and reference DTS images were generated using a shift‐and‐add algorithm^(^ ^23^ ^)^ with a deblurring algorithm applied to selectively remove the imaging noise and overlying structures.^(^ ^24^ ^)^ DTS images contain out‐of‐plane artifact, streaking artifacts, and structure deformation in the beam direction that are not present in the planning CT.^(^ ^23^ ^)^ Unlike CT volumes that are sliced in the axial direction, DTS images with high spatial resolution in the plane oriented perpendicular to the DTS beam line are sliced in the beam direction. The difference in image viewing orientation and image quality complicates direct registration of DTS to CT. Therefore, it was essential to generate a reference DTS volume with image quality similar to that of the treatment DTS.^(^ ^19^ ^)^ To generate the treatment DTS volumes, 2D projections were back‐projected to a set of slices in image space, followed by a 3D deblurring step performed on a subset of the initial back‐projected slices to minimize the appearance of overlying structures in each slice. The reference DTS volumes were generated by reslicing the CT in the treatment DTS direction followed by reprojecting the resliced CT onto a series of DTS slices in image space. Deblurring was then performed on a subset of reprojected slices. Automated DTS volume registrations were conducted through feature matching (cross‐correlation) followed by triangulation. Triangulation of the cross‐correlation match results using two or more short‐arc DTS volumes improved the registration accuracy in the direction along the DTS beam line.

#### B.1 Treatment DTS reconstruction

**Figure 1:**
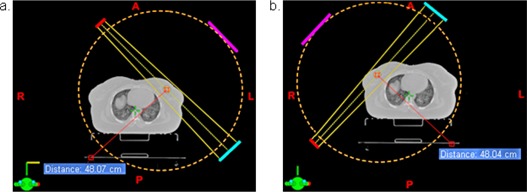
Illustration of the medial tangential arc geometry for imaging the left breast (a) and the lateral tangential arc geometry for the right breast (b). KV source is shown in red, kV imager is shown in blue and gantry head is shown in magenta. The orange dotted line indicates the clearance available for 360° gantry rotation (about 42 cm). Distance of kV imager to isocenter is 50 cm, and distance from kV source to kV imager is 100 cm.

**Figure 2:**
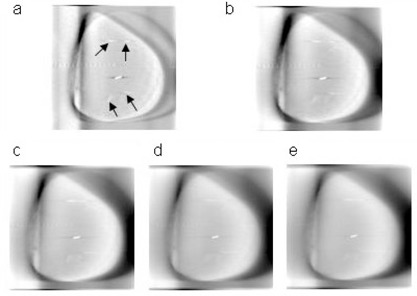
Images produced with different percentages of initial back‐projected slices used for deblurring the DTS algorithm: 20%, 40%, 60%, 80%, and 100%, (a) to (e), respectively. A greater percentage of the initial back‐projected slices used for deblurring produces images with less out‐of‐plane signal leakage into the specific plane under consideration. As indicated by arrows in (a), additional clips located in the adjacent slices were present in the background when using only 20% of initial back‐projected slices for deblurring the DTS algorithm. The artifacts and noise that run through the center of the image from left to right were less pronounced when a larger percentage of slices was used for deblurring.

To reconstruct the treatment DTS images, projections acquired within the range of $\theta_{c}$ – δθ/2, $\theta_{c}$ + δθ/2 were incorporated into the reconstruction; $\theta_{c}$ was the specified middle arc angle (the gantry angle at the center of the arc length specified), and δθ was the DTS imaging arc length. To achieve maximal gantry clearance for imaging, the medial tangential arc was used for imaging of the left breast and the lateral tangential arc was used for the right breast.^(^ ^8^ ^)^ Figure 1 illustrates the beam orientation used with respect to the patient. With the imaging isocenter placed at the treatment isocenter, the gantry head could potentially collide with the couch or patient with a 360° rotation of the gantry. The potential collision could be avoided using a partial rotation, as in the case of CBTS imaging. The middle arc angles are listed in Table 1. The images were reconstructed with 150 mm initial back‐projection depth and $60\;\times \;60\;\times 75\;{\text{mm}}^{3}$ final reconstruction volume. The larger initial back‐projection depth provided additional slices for removal of out‐of‐plane structures by the 3D filtering. The percentage of the initial back‐projection slices used for filtering determined the final DTS image quality. (A greater percentage results in an improved filtering process (Fig. 2) and produces images with smoother background and less overlying structure in the in‐plane image; however, it takes more computational time.) Reconstruction of the DTS images using the procedure described above took 10–20 seconds, depending on the arc length and slice spacing.

#### B.2 Reference DTS reconstruction

The reference DTS images were reconstructed from the resliced planning CT oriented in the DTS beam direction. The resliced CT volume was reprojected onto image space to generate virtual projections (DRR). The subsequent back‐projection, filtering, and deblurring process was performed on the virtual projections to generate DTS volumes. Reconstruction of reference DTS with the final volume of $60\;\times \;60\;\times 33\;{\text{mm}}^{3}$ took 10–30 seconds.

### C. Registration (template matching and triangulation)

DTS image registration was performed in two steps: feature matching based on a user‐defined template, and triangulation of the matched results. For template matching, a target pattern (template) was shifted to each location in the image within the search region $\left(15\;\times \;15\;\times 15\;{\text{mm}}^{3}\right)$. A cross‐correlation match is size‐ and shape‐specific; the similarity of image quality between the two parts of the image pair is important. Therefore, a Gaussian filter was applied to the reference and treatment DTS images before the match to reduce the difference in resolution quality between the paired images. The DTS pair to be matched should be of the same anatomy and must be oriented at the same gantry angle.

The effective slice spacing of a DTS image varies throughout the DTS volume.^(^ ^23^ ^–^ ^26^ ^)^ The effective slice spacing is determined by the scan angle used for image reconstruction. A larger scan angle results in thinner effective slice spacing because of the smaller effective slice thickness.^(^ ^23^ ^,^ ^24^ ^)^ Due to the limited scan angle, a DTS image has suboptimal depth resolution, which results in a less precise match in the depth direction. To improve the registration results, triangulation was performed on the match results from two or more short DTS arcs. As the DTS arcs come from different gantry angles, this provided more spatial information in the depth direction for triangulation.

## D. Evaluation of DTS software

### D.1 Effect of reconstruction parameters on image quality

#### D.1.1 Arc length (δθ)

**Figure 3:**
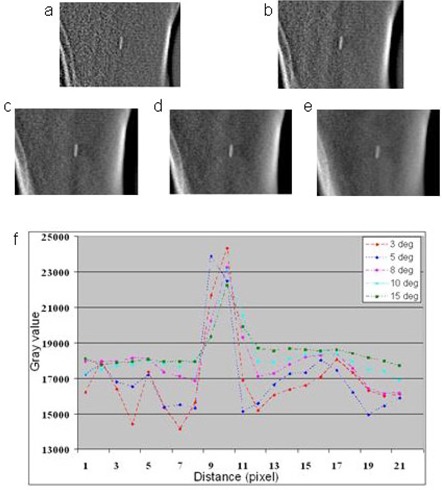
Treatment DTS images reconstructed with the arc length of: 3° (a), 5° (b), 8° (c), 10° (d), 15° (e); corresponding profile plots (f) across the clip in the horizontal direction. Images reconstructed with smaller arc lengths had better contrast but noisier background, while image reconstructed with larger arc lengths appear blurred.

DTS image quality was evaluated as a function of the imaging arc lengths. Figure 3 shows DTS images reconstructed using various arc lengths (3° ≤ δθ ≤ 15°) and the corresponding profile plots across the clip in the horizontal direction. DTS image quality reduces with decreased arc length; images reconstructed from a smaller arc length contain more background noise and deformations in the beam direction. However, the image contrast decreases with increased arc length due to the filtering process applied to each back‐projection. This causes blurring of the images with the arc length δθ < 10° and decreases the registration accuracy.

#### D.1.2 Reconstruction slice spacing

**Figure 4:**
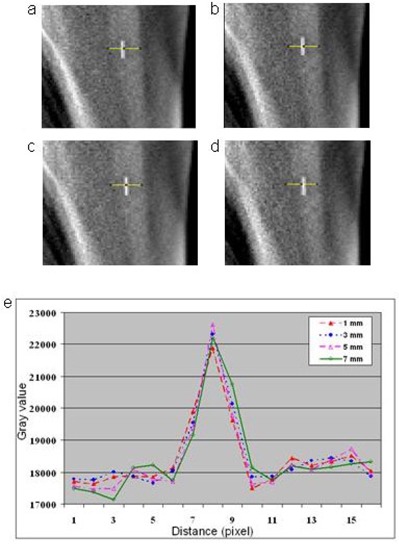
Treatment DTS images reconstructed with arc length of 10° and slice spacing of: 1 mm (a), 3 mm (b), 5 mm (c), and 7 mm (d). Profile plots (e) across the clip in the lateral direction, as indicated by the yellow lines, showed that the image contrast of DTS images did not depend on reconstruction slice spacing.

The effect of reconstruction slice spacing on image quality was evaluated for DTS images reconstructed with 1, 3, 5, and 7 mm slice spacing and 10° arc length. Comparison of the image profiles (Fig. 4) showed no significant contrast and resolution difference in DTS images reconstructed with various slice spacing. Depending on the size and orientation of the clip in the DTS image, a clip appeared in fewer slices in the images with thicker slices. However, this difference was only observed in the in‐plane image, and did not affect the final registration accuracy in the slice depth direction, which depends on the accuracy of the triangulation. On the contrary, the effective slice thickness of DTS images is determined by the scan angle used for image reconstruction. The effective slice thickness varies throughout the DTS volume, and the image quality is degraded by superposition of the structures outside of the focal plane.^(^ ^25^ ^–^ ^27^ ^)^ A larger scan angle results in thinner effective slice thickness because of better slice‐to‐slice resolution.^(^ ^24^ ^)^ In the DTS software, the specified parameter was the spacing between the DTS slices and not the effective thickness of the DTS slice.

#### D.1.3 DTS image registration (rectangular phantom and breast phantom)

Planning CTs of the phantoms were acquired and treatment isocenter position was defined. CBCT projections were acquired with i) the phantom positioned at the isocenter, and ii) the phantom shifted by a known distance. The treatment DTS images generated with and without applied shifts were registered to the reference DTS images created from the CT images. Obtained shifts were compared to the shifts applied. The difference between the found and applied shifts was used to evaluate the accuracy of the registration software.

## III. RESULTS

### A. Registration accuracy dependence on imaging parameters (phantom data)

DTS images with various arc lengths and arc separations were reconstructed for a rectangular phantom. The registration accuracy for an arc separation of 20° or more was within a millimeter (Table 2); however, for an arc separation smaller than 20°, the registration accuracy was 1–2 mm. Larger arc separation improved the registration accuracy only marginally (about 0.3 mm at most), because of the simplicity of the phantom that produced very accurate registration even when using small arc separation. In addition, for arc separation < 20°, the registration accuracy improved for larger arc lengths. The effect of slice spacing on the registration accuracy was not tested on phantom data. The registration performed on the breast phantom produced similar results (not listed here).

**Table 2:** DTS image registration accuracy.

	*Arc Length: 20*°			*Registration Accuracy Arc Length: 10*°			*Arc Length: 5*°		
*Arc Separation (degree)*	*X (mm)*	*Y (mm)*	*Z (mm)*	*X (mm)*	*Y (mm)*	*Z (mm)*	*X (mm)*	*Y (mm)*	*Z (mm)*
90	0.0	0.1	0.7	0.5	0.0	0.1	0.4	0.1	0.4
80	0.4	0.3	0.3	0.2	0.3	0.7	0.1	0.2	0.3
70	0.3	0.1	0.3	0.4	0.2	0.1	0.4	0.0	0.6
60	0.4	0.2	1.0	0.1	0.3	0.2	0.2	0.0	0.4
50	0.1	0.1	0.8	0.3	0.1	0.1	0.2	0.1	0.4
40	0.2	0.2	0.3	0.5	0.1	0.2	0.3	0.1	0.5
30	0.4	0.3	0.4	0.4	0.4	0.5	0.4	0.0	0.4
20	0.5	0.1	0.4	0.5	0.2	0.2	0.2	0.1	0.3
10	1.0	0.3	1.5	1.3	0.4	0.8	1.8	0.3	1.8

Registration accuracy for DTS images of a breast phantom with arc lengths of: 5°, 10°, and 20° and various arc separations: 10°–90°.

### B. Registration accuracy dependence on imaging parameters (patient data)

#### B.1 Arc length (δθ)

**Figure 5:**
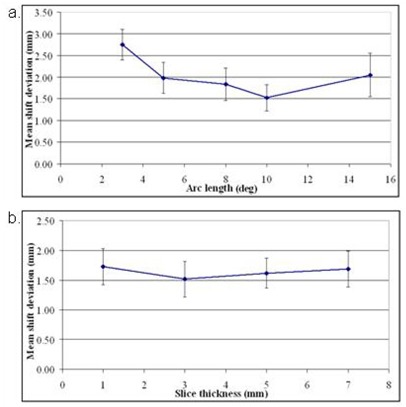
Dependence of registration accuracy on imaging arc length for DTS images reconstructed with slice spacing of 3 mm (a); dependence of registration accuracy on DTS slice spacing for images reconstructed with arc length of 10° (b). Arc separation was 20° for both plots.

The registration accuracy was evaluated on the patient data reconstructed with slice spacing of 3 mm as a function of imaging arc length (δθ = 3°, 5° 8°, 10°, 15°). As shown in Fig. 5(a), the highest registration accuracy obtained was 1.5 mm with a standard deviation σ of 0.6 mm. This was achieved using DTS images reconstructed with the arc length of 10°. For a smaller imaging arc length (e.g., δθ = 3°), an average registration accuracy of 2.8 mm (σ = 0.7 mm) was achieved. Two cases with clips of lower HU (HU ≤ 600 ) failed when registration was performed on images reconstructed using an arc length of 5° or smaller. The cross‐correlation match failed in the two cases performed on images reconstructed using a 3° arc length and clips of low density (HU ≤ 1000 ).

#### B.2 Slice spacing

The registration accuracy dependence on slice spacing was evaluated on DTS images reconstructed with a 10° arc length and a 20° arc separation. The in‐plane image quality (contrast, background noise level, and amount of blurring) was similar for all images reconstructed with various slice spacing. Depending on its size and orientation, a clip was observed in: 13–20 slices (1 mm slice spacing), 6–12 slices (3 mm slice spacing), 4–8 slices (5 mm slice spacing), and 2–5 slices (7 mm slice spacing) in the DTS image.

As shown in Fig. 5(b), the mean registration accuracy of the DTS images reconstructed with slice spacing of 1–7 mm was between 1.5 mm and 1.7 mm (σ=0.6 mm). It needs to be noted that the final registration accuracy was a combined measure of both cross‐correlation and triangulation steps. While the results from a cross‐correlation match alone were inferior to the combined registration results, there was no obvious dependence of the cross‐correlation match on the slice spacing. However, reconstruction of the same volume with smaller slice spacing required longer time. The DTS image reconstruction time increased by up to 15 seconds when the slice spacing was reduced from 3 mm to 1 mm.

#### B.3 Arc separation $\left(\theta_{c1}-\theta_{c2}\right)$

Theoretically, registration accuracy improves with an increase in separation between the two arcs, provided that the cross‐correlation match results are accurate for each arc. However, clip orientation and distribution were different in each arc orientation; some clips overlapped with each other or with the ribs in the beam's eye view (BEV). This induced errors and failures in the cross‐correlation match and led to inaccurate triangulation. Table 3 shows the registration accuracy as a function of arc separation for DTS images reconstructed with 3 mm slice spacing and 10° arc length. In general, a 20° arc separation was sufficient for accurate registration; however, the selected arcs need to provide good visibility of at least two well‐separated clips. Arc separation of 10° was possible, but did not always produce reliable triangulation.

**Table 3:** Dependence of registration accuracy on DTS arc separation.

	*Dependence of Registration Accuracy on Arc Separation*					
	*Patient Data (Left Breast)*			*Patient Data (Right Breast)*		
*Arc Separation (degree)*	*X (mm)*	*Y; (mm)*	*Z (mm)*	*X (mm)*	*Y (mm)*	*Z (mm)*
10	2.9	1.1	6.8	4.7	0.5	0.5
20	1.7	0.5	0.0	0.7	0.8	2.1
30	1.3	0.7	0.5	2.7	0.1	0.3
40	Cross‐correlation failed			Cross‐correlation failed		
50	Cross‐correlation failed			3.0	0.3	0.3
60	0.7	1.0	1.7	Cross‐correlation failed		
70	1.1	0.5	0.0	2.4	0.1	0.2
80	0.9	1.0	0.8	0.2	0.9	0.1
90	0.8	1.1	1.1	1.8	0.7	0.2

Registration results using two short‐arc DTS images reconstructed with 10° arc length. The results reported are for a single patient in each case. The registration accuracy has no apparent dependence on the arc separation. However, visibility of the clips in a particular arc orientation played an important role in ensuring accurate registration.

#### B.4 Distribution, number, and characteristics of clips

**Figure 6:**
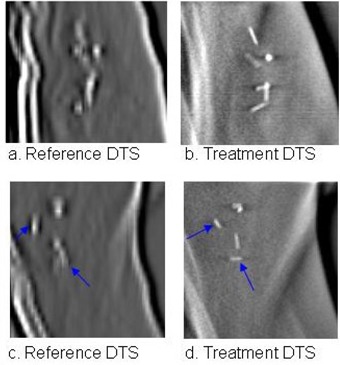
Example of inferior clip distribution, ((a) and (b)); some clips overlapped in the reference images, causing failure in cross‐correlation match; example of well distributed clips, ((c) and (d)). As indicated by the arrows, some of the clip orientations were different in reference and treatment DTS.

Distribution of surgical clips is a critical factor for accurate registration in DTS images. In the reference DTS images, clips distributed close to each other (e.g., < 3 mm apart) appeared as a big cluster of bright pixels due to the structure deformation, resulting in inaccurate registration. Figure 6(a) shows six closely distributed clips overlapped in the reference DTS images. These clips resembled the ribs and led to a false cross‐correlation match. On the other hand, a smaller number of well distributed clips (as in Figs. 6(c), 6(d)) are more favorable for registration. Smaller clips with lower HU had lower contrast and resulted in less accurate registration. We have determined that clips with HU ≥ 1000 are optimal for accurate registration. Clips with lower HU (HU = 500−800) are possible to be registered for the DTS images reconstructed but require larger arc length (δθ ≥ 8°).

#### B.5 Number of arcs

DTS images are considered as pseudo‐3D volumes due to their suboptimal depth resolution. Registration of a DTS image pair from a single arc produced less satisfactory results. An increased number of DTS pairs used in registration provided more spatial information in the slice depth direction for triangulation, thereby improving the final registration result. However, careful selection of arc orientations was crucial because the cross‐correlation match on DTS images with overlapped clips and structures might fail. An optimal arc orientation could be selected at the CT planning stage to avoid the angles which are likely to result in a clip–clip or clip–rib overlap. When arcs were properly selected, the registration of two arcs with an arc separation of 20° was sufficient to provide an accurate registration (e.g., better than 1 mm). All the registration results listed above were done with only two short DTS arcs.

## IV. DISCUSSION

This study presents a thorough evaluation of the nonclinical software provided by Varian to reconstruct and register DTS images for patient setup using the clinically available OBI system. If clinically available, this software would provide a viable alternative to CBCT imaging and potentially solve some of the challenges currently associated with CBCT imaging, especially when used for breast cancer patient setup. In particular, it would allow dose reduction to organs at risk, minimize collision situations, and shorten the setup time. When using this software, the time required for DTS reconstruction was 10–30 seconds, while the registration time was less than 20 seconds. In comparison, CBCT scan time is typically over one minute.^(^ ^28^ ^)^ CBCT images reconstruction time is between 1–2 minutes^(^ ^22^ ^)^, depending on the reconstruction parameters chosen (i.e., obtaining highest image quality or fastest reconstruction speed). The automated registration of CBCT images in Offline Review takes less than 20 seconds. The time for the whole process utilizing CBCT is 3–4 minutes.

As this software heavily relies on an automated clip localization process, it therefore strongly depends on the correlation (resemblance) between the reference and treatment DTS images and on the region of interest (ROI) used for registration. The streaking artifacts originating from the high‐density clips might lower the correlation between the images and result in less accurate registration. In our study, both the reference and treatment DTS images were generated with the same acquisition and reconstruction parameters; therefore, they have comparable image quality. In addition, the cross‐correlation match was performed on the in‐focus plane slice where the artifacts from the out‐of‐focus plane objects were minimal. The feature matching used the correlation of the whole ROI (or template) chosen for registration, therefore all features (clips, soft tissue, or ribs) included within the template were used for registration. The effect of individual seed migration on the positioning accuracy was not evaluated. However, we observed that for the cases with closely distributed clips, the correlation between the two images was lower, and the registration results were less accurate.

In this study, we described how to select the target template and DTS imaging geometry to achieve appropriate image quality for given clips characteristics. The following guidelines for selecting the reconstruction and registration parameters may serve as a means to produce reliable registration results.

### A. Number of clips

The number of clips used for registration was not the most determining factor for an accurate registration. Figure 6 shows that a large number of closely distributed clips might induce errors in the cross‐correlation match. We determined that a DTS image with three or more well‐separated clips (3 mm apart) in a selected imaging beam's eye view would provide an accurate registration.

### B. Clip distribution

Well‐distributed clips are defined as being separated by a few pixels in a particular BEV. Due to possible clip migration and breast deformation, the clips may appear in a different position and orientation in the reference and treatment DTS images. Figure 6 shows two examples of favorable and unfavorable clip distributions. In the case of a large amount of closely distributed clips (e.g., < 3 mm in the BEV), the difference in clip orientation may lower the correlation between the reference and treatment DTS.

### C. Middle arc angle based on treatment sites

Medial arcs were used for registration of the left breast cases, while lateral arcs were used for the right breast cases. Two short DTS arcs with 20° arc separation were used for all image registrations. For the left breast, all cases were reconstructed with the arc orientation in the range of 305°–335°, while a range of 205°–265° was used for the right breast cases.

### D. Clip distance from ribs and breast surface

Ribs and breast surface with high contrast in DTS images caused a large degree of bias in clip‐based registration. Volumes reconstructed from the beam angles with a BEV that is close to these areas should be avoided. In general, DTS images where the clips are located at least 1 cm away from the ribs and breast surface in the BEV appear to be optimal for registration.

## V. CONCLUSIONS

Nonclinical Varian DTS software for DTS image reconstruction and registration was evaluated and found to provide setup accuracy within 1 mm when optimal imaging geometry was selected. Optimal data acquisition parameters were determined for DTS imaging for breast cancer patient setup. At least two arcs were necessary for acceptable accuracy of setup evaluation. An optimal arc separation was found to be 20°, and an optimal arc length was found to be 10°. The slice spacing of the DTS reconstruction was not a factor in the clip‐based registration. Accuracy of 2 mm or less was achieved in most cases when reconstruction arc length was 5° or larger and clip density was at least 1000 HU.

## ACKNOWLEDGMENTS

This research was funded in part by a grant from Varian Medical Systems Inc. and a Kaye Scholars Grant.
